# The cycle of seagrass life: From flowers to new meadows

**DOI:** 10.1002/ece3.10456

**Published:** 2023-08-31

**Authors:** Gary A. Kendrick, Marion L. Cambridge, Robert J. Orth, Matthew W. Fraser, Renae K. Hovey, John Statton, Charitha B. Pattiaratchi, Elizabeth A. Sinclair

**Affiliations:** ^1^ School of Biological Sciences and UWA Oceans Institute The University of Western Australia Western Australia Crawley Australia; ^2^ Virginia Institute of Marine Science College of William and Mary Gloucester Point Virginia USA; ^3^ Oceans Graduate School and UWA Oceans Institute The University of Western Australia Crawley Western Australia Australia

**Keywords:** clonal diversity, flowering, microsatellite DNA, *Posidonia australis*, recruitment, reproductive effort, seed predation

## Abstract

Understanding sexual reproduction and recruitment in seagrasses is crucial to their conservation and restoration. Flowering, seed production, seed recruitment, and seedling establishment data for the seagrass *Posidonia australis* was collected annually between 2013 and 2018 in meadows at six locations around Rottnest Island, Western Australia. Variable annual rates of flowering and seed production were observed among meadows between northern and southern sides of the island and among years. Meadows on the northern shore consistently flowered more intensely and produced more seeds across the years of the survey. Inter‐site variation in clonal diversity and size of clones, seed production, wind and surface currents during pollen and seed release, and the large, but variable, impact of seed predation are likely the principal drivers of successful recruitment into established meadows and in colonizing unvegetated sands. The prolific but variable annual reproductive investment increases the probability of low levels of continuous recruitment from seed in this seagrass, despite high rates of abiotic and biotic disturbance at seedling, shoot, and patch scales. This strategy also imparts a level of ecological resilience to this long‐lived and persistent species.

## Graphical abstract

Flowering, seed production, seed recruitment, and seedling establishment for the seagrass *Posidonia australis* was collected annually between 2013 and 2018. Meadows on the northern shore consistently flowered more intensely and produced more seeds across the years of the survey. Variation in clonal diversity and size of clones, seed production, island wakes developed by wind and surface currents, and the large, but variable, impact of seed predation are likely the principal drivers of successful recruitment from seeds.

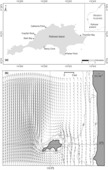

## 1 INTRODUCTION

Sexual reproduction drives the evolutionary persistence of flowering plants through maintaining genetic diversity and connectivity that contribute to demographic and evolutionary processes (Harper et al., 1977; Kendrick et al., 2017). Understanding these processes is important for conservation, management, and restoration of seagrasses in disturbed environments (Statton et al., 2018; Waycott et al., 2009). Detailed studies of seagrass sexual reproduction described dispersal (e.g. Ruiz‐Montoya et al., 2015), genetic connectivity (e.g. Sinclair et al., 2016, 2018), and bottlenecks to seedling recruitment (e.g. Statton et al., 2017). However, there is no information on flowering, seed production, and long‐term survival of seedlings to complete the cycle.

Seagrasses are highly clonal marine angiosperms that make significant investments in sexual reproduction (Kendrick et al., 2017; Sherman et al., 2018). *Posidonia australis* is an Australian endemic, monoecious seagrass species with hermaphrodite flowers that are protandrous, where anthers mature, and pollen is released before the stigma develops on the female flower. Mating system studies indicate that *P. australis* is 100% outcrossed and selfing results in ovule abortion (Sinclair, Gecan, et al., 2014). Thus, the limitation of pollen dispersal and successful fertilization of flowers is clonal size and diversity (Sinclair et al., 2020). This species produces abundant flowers from July to August, across much of its 4200 km geographical range and fertilized ovules develop into seeds within a spongy fruit (pericarp) for approximately 3 months until their release between October and December (early Austral Summer). These positively buoyant fruit contain a single precocious developing seed and can disperse over 10 s −100 kilometers, although most seed release occurs within 1 s–100 s of meters of parents, resulting in relatively open populations in *P. australis* where connectivity is influenced by local and wind‐driven surface currents, wind, and geographical barriers (Ruiz‐Montoya et al., 2015; Sinclair et al., 2018; Sinclair, Krauss, et al., 2014).

Once seeds of *P. australis* dehisce from their floating fruit and settle onto suitable habitat, they undergo significant seed predation that can remove ≤80% of seeds daily in seagrass meadows, or 10% daily over bare sand suggesting seeds are more likely to recruit into gaps within existing meadows (Orth et al., 2006; Statton et al., 2017).

Seagrass recruitment has been assessed for some seagrass species (Kendrick et al., 2017), but most studies have failed to quantify seedling recruitment and survival rates across multiple years. A recruiting seedling is considered to be the most sensitive stage of the plant life cycle (Eriksson & Ehrlén, 1992; Harper et al., 1977), is a bottleneck and therefore subject to the highest mortality rates (Orth et al., 2006; Statton et al., 2017). In the European seagrass, *Zostera noltii*, recruitment accounted for nearly 20% of the new shoots each spring and ~30% were traced to seeds from up to 3 years earlier, suggesting that the stability and genetic diversity of meadows is related to persistent seed banks (Zipperle et al., 2009). Seeds of *Posidonia* species are recalcitrant, developing directly without dormancy, and so do not form persistent seed banks. Survival of seedlings after seeds settle and become established has been reported to be as high as 66% over 3 years for *Posidonia oceanica* in the Ligurian Sea, Italy (Balestri et al., 1998). Quantifying survival rates of seedlings is necessary for understanding the resilience of seagrasses, but also it gives insight into post‐settlement factors that limit successful recruitment, such as storm disturbance and predation, which can lead to improved management and more success in seed‐based restoration.

The specific objectives in this paper are to characterize the contemporary demographic processes of sexual reproduction: determine flowering, reproductive effort, fertilization success, seed production, seed predation, and seedling survival. We interpret these demographic processes with the aid of oceanographic and genetic data.

## 2 METHODS

Rottnest Island is an oligotrophic marine environment with genetically diverse *P. australis* meadows both around the island and in the nearby Perth coastal marine region. This paper examines multiple spatial data sets on all aspects of the flowering process accrued over a period of 6 years (2013–2018) at Rottnest Island, Western Australia, patterns of seed predation over 12 years (2003–2014) and recruitment into juxtaposed unvegetated sand habitats over 5 years (2014–2018).

### 2.1 Study location and oceanographic features

**FIGURE 1:**
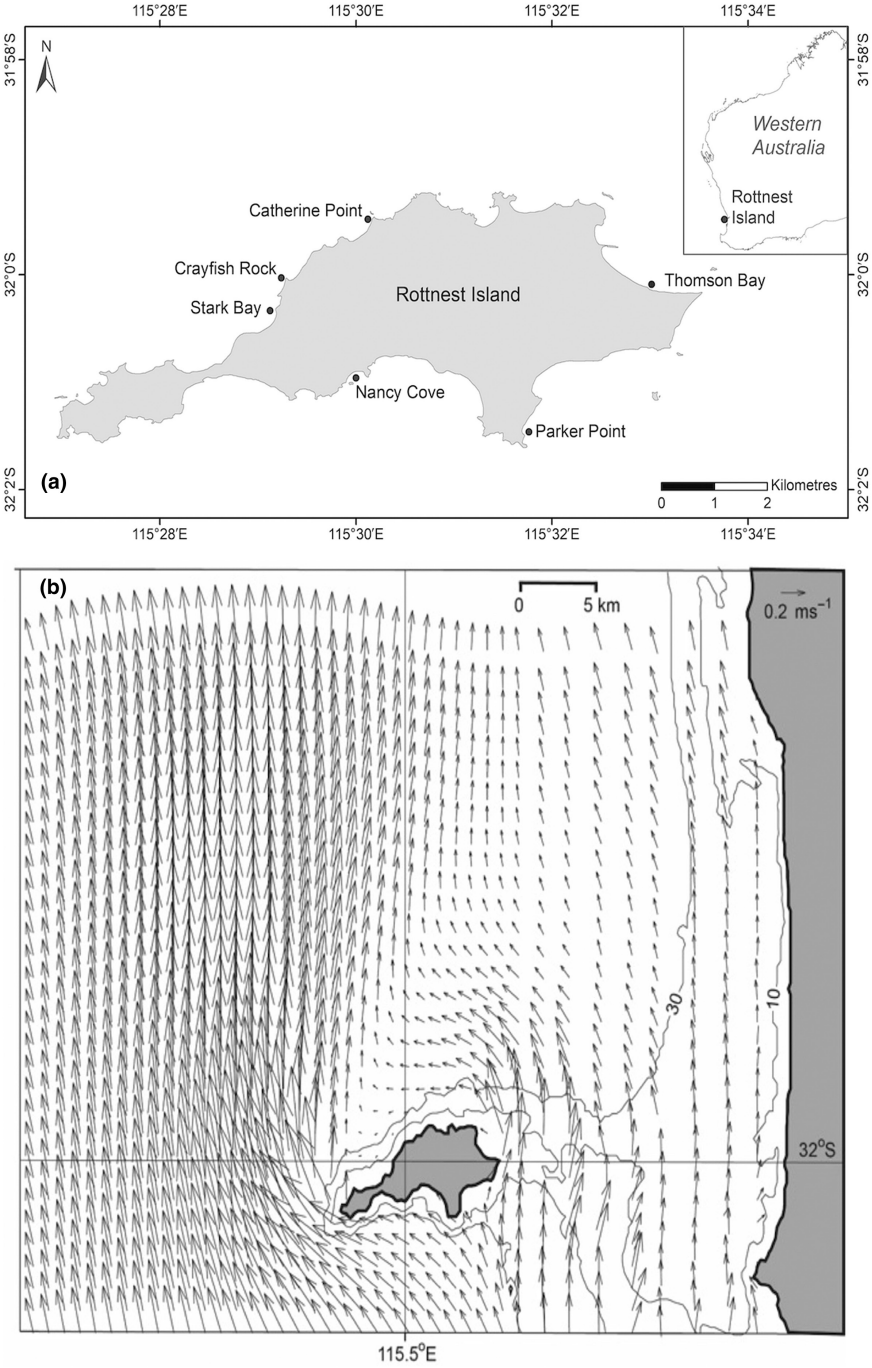
Location of Rottnest Island, Western Australia showing (a) sampling locations and (b) Summer surface currents in the Rottnest Island region resulting from southerly winds derived from a numerical model presented by Alaee et al. (2007). Bathymetry in m.

Rottnest Island (S 32.000625°, E 115.548531°) (Figure 1a) is 10.5 km long and 4.5 km wide, located on the relatively flat continental shelf, ~20 km off the West Australian coastline (Figure 1b). The location of the island is such that it acts as barrier for alongshore water flow along the shelf. During the austral summer months (November to March), southerly winds dominate the region that is augmented by strong southerly sea breezes (Rafiq et al., 2020). The northward flowing Capes Current (Pearce & Pattiaratchi, 1999) and the southward flowing Leeuwin Current (Cosoli et al., 2020) are the dominant currents that influence the Rottnest Island region during summer and winter, respectively. Interannual variation in southerly winds in temperate Western Australia strongly influences surface currents, and the cycles of ENSO (El Niño Southern Oscillation) add interannual variability to wind intensities. The period 2010–2011 was an extreme local marine heatwave driven by a strong La Niña that influenced marine temperature and biota until 2013, followed by El Niño's between late 2014 to early 2016, and mild La Niña conditions between late 2016 and 2018. Sea surface temperatures range approximately 4°C, from 19 to 23°C. Sea Surface monthly temperature anomalies have declined from approximately +0.5°C in 2013 to −1°C in 2015 (Pattiaratchi & Hetzel, 2020).

### 2.2 Shoot density, inflorescence density, and seed production

Investment in sexual reproduction, vegetative shoots, inflorescences, flower and fruit (seed) densities were determined within *P. australis* meadows among six locations Rottnest Island: Parker Point and Nancy Cove on the southern shore, Stark Bay, Crayfish Rock and Catherine Point on the northern shore and Thomson Bay on the eastern shore (Figure 1a). Sampling was performed before fruit release in November between 2013 and 2018. Vegetative shoot density in seagrass meadows was assessed using 10 × 0.04 m^2^ randomly sampled quadrats. Inflorescence density was assessed from five replicate 10 × 1 m (10 m^2^) belt transects. Flower and fruit (seed) production per inflorescence were determined from the random collection of 12 inflorescences from transects at each site. Inflorescences consisted of a stem (petiole) bearing several spikes (3–12) with 3–5 hermaphrodite flowers on each spike. Following successful pollination, fruits develop for approximately 12 weeks. For each inflorescence, the number of fully developed fruit, undeveloped fruit and remains of flowers that had not been pollinated were counted on each spike. Total number of flowers per inflorescence were derived from the sum of all fruit, undeveloped fruit and remains of flowers. Seed to ovule ratio was determined from the total number of mature fruit (1 fruit = 1 seed) divided by the total numbers of flowers (1 flower = 1 ovule).

We ran a GLMM using the R package lme4 to statistically analyze flowering data (Bates et al., 2015). A log Poisson generalized linear mixed‐effects model using Laplace approximation of maximum likelihood was fitted to inflorescence densities and shoot densities. A negative binomial generalized linear mixed‐effects model using Laplace approximation of maximum likelihood was fitted to flower and seed (fruit) densities. The best fitted GLMM for inflorescence densities and shoot densities was response variable ~ location × year + (1|transect) + (1|observation effect). The variable (1|observation effect) was added to address overdispersion in this data (Harrison, 2014). The best fitted GLMM for flower and seed (fruit) densities was response variable ~ location × year + (1|rep inflorescence). Analysis of deviance was used to evaluate each model term.

### 2.3 Clonal diversity in meadows and recruits

Fifty adult *P. australis* shoots were collected from randomly generated GPS coordinates within a 50 m diameter circle from four meadows around Rottnest Island: Thomson Bay (RTB), Stark Bay (RST), Nancy Cove (RNC), and Parker Point (RPP) (Figure 1). Approximately, 30 1‐year‐old *P. australis* seedlings were also collected for genotyping following annual surveying for two consecutive years (2014 and 2015). All adult shoot and seedling samples were genotyped for seven polymorphic DNA loci (*Pa*A1, *Pa*A105, *Pa*A120, *Pa*B6, *Pa*B8, *Pa*B112, and *Pa*D113). Laboratory and genotyping methods are described in full in Sinclair, Krauss, et al. (2014).

Clonal and genetic diversity indices were estimated for all adult shoots and seedling cohorts using genalex version 6.5 (Peakall & Smouse, 2012). The “Find Clones” option was used to identify shared multilocus genotypes (MLGs) within and among meadows. P_ID_ was then used to determine whether shared MLGs were considered to come from the same vegetative clone or a separate recruitment event (from seed). Clonal richness (*R* = (*G*−1)/(*N*−1)), where *G* = number of MLGs, and *N* = number of samples, was estimated for each meadow (Dorken & Eckert, 2001). Genetic diversity parameters were based on the complete data set: the total number of alleles (Na), number of private alleles (*p*[i]) estimated as those alleles that only occur in a single sampled meadow, observed (*H*_o_) and expected heterozygosity (*H*_e_), and Fixation Index (*F*) using genalex. Tests for Hardy–Weinberg equilibrium (HWE) were conducted using GENEPOP v4.2 (http://genepop.curtin.edu.au/).

### 2.4 Seed predation

Seed predation was assessed over two distinct time periods between 2001 and 2016 (2001, 2003, 2004, and then again in 2013, 2014, 2016) at Parker Point to determine whether predators may limit seed survival. Full details on the tether and seed board methods can be found in Orth et al. (2006) and Manley et al. (2015), respectively. In 2001, 2003, 2004, 10 tethered seeds were placed in a continuous meadow of *P. australis*, approximately 2 m apart and 1–2 m from the sand edge, as well as in sand also 1–2 m from the seagrass meadow. In 2013, 2014, and 2016, seed boards containing three seeds each were used at three sample locations spaced approximately 20 m apart. At each of the three locations, three seed boards were placed 3 m from each other at 1–2 m from the sand‐seagrass edge in each habitat. Seeds were assessed for signs of predation every 24 h for 7 days and replaced if missing or partially eaten. The experiment was conducted for only 3 days in 2016 due to weather and logistical constraints. A binomial generalized linear mixed‐effects model using Laplace approximation of maximum likelihood was fitted to seed presence/absence data. We included the habitat and year during which seed predation was evaluated as interacting fixed effects within the model. The survey method used (seed tether or seed board) was treated as a random effect (R package lme4). Analysis of deviance was used to evaluate each model term.

### 2.5 Recruitment of seedlings

To quantify seedling recruitment and survivorship, we surveyed sparsely vegetated, 300 m^−2^ plots adjacent to well‐established *P. australis* meadows to track the emergence and survival of recruiting seedlings through time. Seedlings are morphologically different from regrown shoots for the first 2–3 years of their life, being smaller, more linear in habit and less robust. If there was any doubt about an individual being a seedling we did not measure it. We counted and phototagged all seedlings at Parker Point, Stark Bay and Thompson Bay in November 2014 and marked their position with a Garmin GPS with a position accuracy of approximately 50 cm (Garmin Corp. Models GPSmap78 and etrex20). We also counted the number of shoots for each seedling to determine shoot and age‐based recruitment. We returned March, 2015, just prior to winter storms, which are known to cause mortality in recruiting seedlings (Statton et al., 2017), to recount and measure all seedlings, including the new 4‐month‐old 2014 cohort. We repeated this process in November 2015–2018, and March 2016–2018.

The vegetative shoots of *P. australis* typically branch once a year in spring (Cambridge & Hocking, 1997), so we constructed histograms of seedling/patch shoot number to approximate the age of each observed seedling/patch. If conditions were favorable, vegetative shoots of *P. australis* may branch for a second time in a year during fall (autumn). We present only the maximum potential age of recruits (one branching event per year). An alternative approach is to assume a much higher branching rate in recruits than adults, but our observations in this study suggest that younger age classes (1–3 years old (yo)) only doubled in shoot number each year. We then pooled the static life tables constructed across years at each location to calculate mean cohort survivorship curves for each location. We utilized 4 years of data for 4‐month‐old recruits (March 2014–2018) and 5 years of data for 12, 24, 36, 48, 60, and 72 months cohorts (November 2013–2018). The survivorship curves are therefore average curves of proportional survival of age classes for the 5 years from static life tables. By pooling our data at each location over the 5 years of the survey, we assume that the relative proportions of all age cohorts were similar across years.

Nonlinear regression was carried out using the power function:

$$y=a\times x^{b}$$

where *y* = proportion of age cohort in total population; *a* = proportion of initial recruits in 2014; *x* = cohort age (months), and *b* = mortality rate. Nonlinear regression statistics for model fit, pseudo‐*R*‐squared, and 95% confidence intervals were calculated in *R* (nlme and nltools in tidyverse package).

### 2.6 Origin of seedlings

A Bayesian population assignment method was used to determine whether seedlings were recruited locally or from a nonlocal meadow using GeneClass2 (Piry et al., 2004). Simulations suggest that 100% correct assignments can be made using Rannala and Mountain's (1997) assignment method with sample sizes of 30–50, 10 microsatellite loci, and an *F*_ST_ = 0.1 (Waples & Gaggiotti, 2006). These parameters were closely met by our data for established meadows (49–50 shoots per meadow, 7 polymorphic loci, and an overall estimate of *F*_ST_ = 0.143). We used data from our mating system study at Parker Point and Stark Bay where the maternal parent was known (Sinclair et al., 2020) to test the power of assignment tests and set confidence thresholds to assign seedlings to a local or nonlocal meadow of origin. “Seeds of known origin” were assigned to one of the four sampled meadows. The probability of a meadow being a seed source (by cohort and individual) was established using the Monte Carlo re‐sampling algorithm using 10,000 replicates resampling permutations following Paetkau et al. (1995). The log‐likelihood (−log(L)) of originating from each source meadow was then calculated following the Bayesian‐based method (Rannala & Mountain, 1997). The proportion of correctly assigned seeds was used to set two assignment thresholds, one based on the correct assignment of the cohort (all seeds) to the meadow of origin, and a second for the correct assignment of individual seeds within each cohort to the meadow of origin. The thresholds reflect a compromise between adopting too low a stringency level, which increased the risk of an incorrect assignment due to genotyping errors and/or inclusion of interpopulation hybrids resulting from pollen flow (see Roques et al., 1999), or too high a stringency level in which too few individuals were assigned. We then applied the threshold to our seedling. An underlying assumption for a cohort assignment is that all seedlings sampled within a cohort originated from the same source meadow. An unambiguous assignment was accepted when the “% score” was above the confidence threshold set using “seeds of known origin”. A cohort or individual that was assigned above the defined threshold to a meadow other than where it was sampled from was regarded as successful recruitment following a dispersal event.

## 3 RESULTS

### 3.1 Oceanographic patterns

During the summer when fruits are released at Rottnest Island, the northward flowing Capes current, transports cooler water, flows around the Island creating an island wake with the strongest currents along the western tip of the island (Figure 1b). This results in weaker currents in the north of the island in the wake region (Alaee et al., 2007). In contrast, stronger southward flow from the Leeuwin Current during winter when pollination occurs results in a re‐circulation eddy and wake region to the south of the island. Shallow waters to the east of the island (<10 m) results in the majority of water flowing along the shelf are deflected around the western end of the island.

The strength of surface currents was heavily influenced by the interannual variation in predominantly south westerly winds during summer (seed release), and was weaker during 2013–2014, stronger between late 2014 and early 2016 (El Niño), and weaker between late 2016 and 2018 (mild La Niña).

### 3.2 Inflorescence density, vegetative shoot density and reproductive effort

**FIGURE 2:**
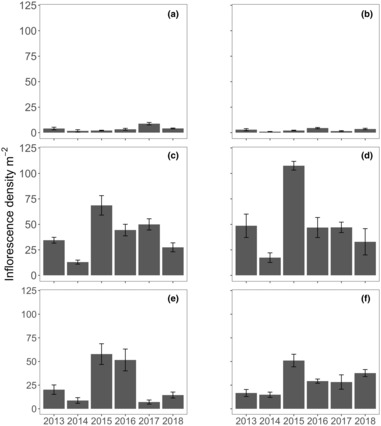
Inflorescence densities (m^2^ ± SE, *n* = 10) measured annually in November from 2013 to 2018 for the six locations: (a) Parker Point, (b) Nancy Cove, (c) Stark Bay, (d) Crayfish Rock, (e) Catherine Point and (f) Thomson Bay at Rottnest Island.

Inflorescence densities varied significantly among locations and across years (Figure 2; Table 1). There was a significant interaction between location and year (Table 1) and overall southern locations were up to 10 times lower in inflorescence densities (Parker Point and Nancy Cove) than other locations on the northern and eastern shores. Interannual variation was significant for all locations, but not consistent among locations (Table S1). Some general patterns were evident, inflorescent densities tended to be lowest in 2014 and highest in 2015, and the locations Catherine Point/Thomson Bay and Stark Bay/Crayfish Rock showed similar interannual patterns.

**TABLE 1:** Analysis of Deviance (Type III Wald Chi‐square tests) for the main response variables (vegetative) shoot density, inflorescence density, flower density, and seed density (individual m^−2^).

Model = Poisson (log)						
Source	Shoot density			Inflorescence density		
	*df*	ChiSq	*p*(>chisq)	*df*	ChiSq	*p*(>chisq)
(Intercept)	1	8560.2	<2.2e−16	1	218.2	<2.2e−16
Location	5	1300.0	<2.2e−16	5	134.8	<2.2e−16
Year	5	25.34	.00012	5	41.97	5.966e−08
Location × year	25	98.55	1.10–10	25	119.7	2.527e−14

Model = negative binomial (log)						
Source	Flower density			Seed density		
	*df*	ChiSq	*p*(>chisq)	*df*	ChiSq	*p*(>chisq)
(Intercept)	1	2334.5	<2.2e−16	1	209.6	<2.2e−16
Location	5	591.9	<2.2e−16	5	199.5	<2.2e−16
Year	5	99.57	<2.2e−16	5	24.88	.000147
Location × year	24	621.8	<2.2e−16	24	289.1	<2.2e−16

*Note*: All *p* << .0001 or “***”.

Vegetative shoot densities varied from <300 to >750 shoots m^−2^, with significant location and interannual variation (Table 1, Figure S1). In general, 2018 had higher shoot densities, while 2013 and 2014 tended to be lower, except for Stark Bay in 2013 (Table S1).

Variation in reproductive effort (inflorescence density/shoot density, mean ± SE, *n* = 10) (Figure S2) generally mirrored the variability in inflorescence density in meadows. Inflorescences accounted for less than 12.5% of the density of vegetative shoots and ranged from <0.5% to >10%.

### 3.3 Flower and seed production

**FIGURE 3:**
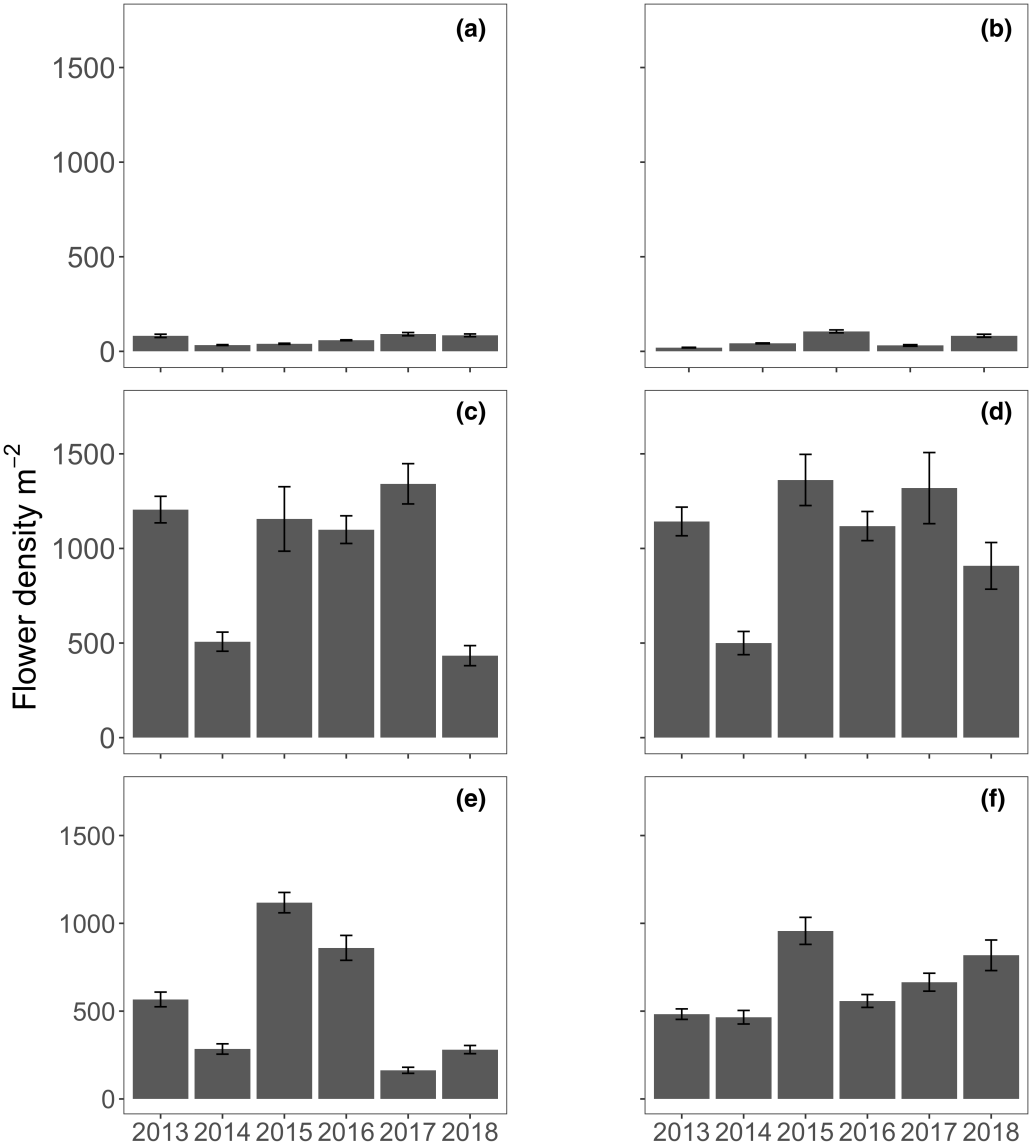
Flower densities (m^2^ ± SE, *n* = 10) measured annually in November from 2013 to 2018 for the six locations: (a) Parker Point, (b) Nancy Cove, (c) Stark Bay, (d) Crayfish Rock, (e) Catherine Point and (f) Thomson Bay at Rottnest Island.

**FIGURE 4:**
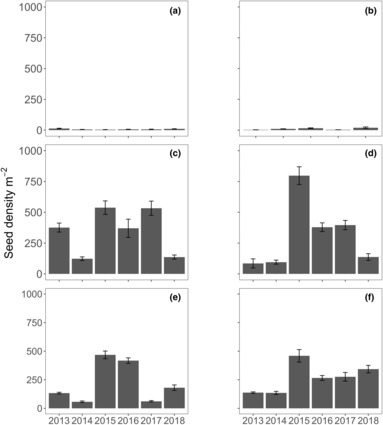
Seed densities (m^2^ ± SE, *n* = 10) measured annually in November from 2013 to 2018 for the six locations: (a) Parker Point, (b) Nancy Cove, (c) Stark Bay, (d) Crayfish Rock, (e) Catherine Point and (f) Thomson Bay at Rottnest Island.

The number of flowers produced by each inflorescence varied between 6 and 60, while the number of seeds varied between 0 and 18. The production of flowers and seeds per square meter varied between 11 and 2540, and 0 and 1182, respectively (Figures 3 and 4). Flower densities on the northern and eastern locations, like inflorescent densities, were generally 10 times higher than the southern locations. Significant differences in flower densities were found with the interaction of location and year (Table 1). Pairwise interactions indicated that flower densities at Stark Bay and Crayfish Rock were relatively similar in 2013 and 2015–2017 (Table S1). Flower densities were consistently higher at Nancy Cove, Catherine Point, and Thomson Bay in 2015, but not for Parker Point. Relative to the other years, 2014 was generally an unsuccessful reproductive year for most locations.

Significant differences in seed density were found in the interaction of location and year (Table 1). Significant pairwise interactions were also observed in seed densities among locations and years (Table S1), with seed density generally low across sites in 2014 and higher in 2015. Flower and seed production numbers varied with the number of inflorescences, although it was evident at some meadows (e.g. Crayfish Rock) that there were higher densities of flowers and a low density of seed produced in 2013 (Figures 2 and 3).

**FIGURE 5:**
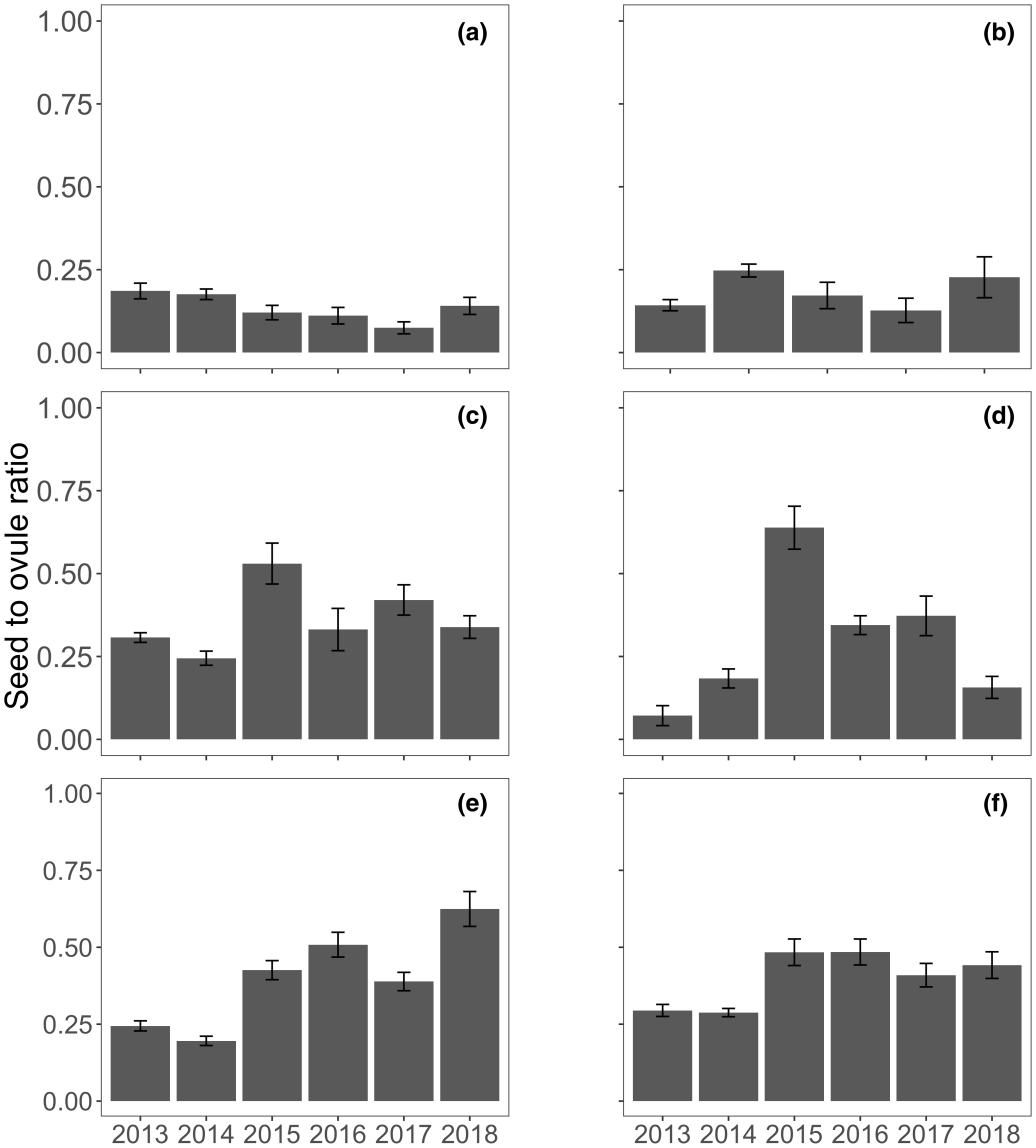
Seed to ovule ratio (mean ± SE, *n* = 10) measured annually in November from 2013 to 2018 for the six locations: (a) Parker Point, (b) Nancy Cove, (c) Stark Bay, (d) Crayfish Rock, (e) Catherine Point and (f) Thomson Bay at Rottnest Island.

Successful fertilization of flowers varied significantly in proportion from 0.08 to 0.62 among locations and years (seed to ovule ratio) (Figure 5). Locations on the southern shores, Parker Point (seed to ovule ratio: 0.08–0.18) and Nancy Cove (0.13–0.15) had fewer seeds produced from flowers than locations to the north and east, Stark Bay (0.25–0.52), Crayfish Rock (0.08–0.62), Catherine Point (0.20–0.51), and Thomson Bay East (0.24–0.48) across all years.

### 3.4 Clonal diversity in meadows and seedlings

Fertilization success, thus overall seed production, may reflect differences in clonal richness among bays at Rottnest (Table 2). Clonal richness for the southern bays, Parker Point (RPP) (*R* = .19) and Nancy Cove (RNC) (*R* = .24) were low with high levels of clonality in the meadows. Clonal richness was greater in Stark Bay (RST) on the northern shore (*R* = .76) and Thomson Bay (RTB) on the eastern shore (*R* = .63). Genetic diversity was also higher in Stark Bay and Thomson Bay than Parker Point and Nancy Cove (Table 2). All meadows were in Hardy–Weinberg equilibrium after removal of clones. All seedling recruits had unique multilocus genotypes, with two exceptions at Stark Bay – one MLG was shared between two recruits in different years and a 2013 recruit had the same MLG as a single shoot.

**TABLE 2:** Genetic diversity indices for established *Posidonia australis* meadows and annual seedling recruit cohorts at four sites around Rottnest Island, Western Australia.

Sample location	Abbrev.	*N*	MLG	*R*	Na	*p*[I]	*H*o	*H*e^a^	*F*	Publication
Mature meadows										
Parker Point (2009)	RPP	49	10	.19	21	6	0.616	0.454*	−0.338	Sinclair, Krauss, et al. (2014)
Nancy Cove (2015)	RNC	50	13	.24	21	0	0.491	0.383*	−0.210	This study
Stark Bay (2015)	RST	50	38	.76	33	0	0.549	0.523	−0.053	This study
Thompson Bay (2015)	RTB	50	32	.63	35	6	0.424	0.436*	0.065	This study
Seedling recruits: cohorts by year of seed production										
Parker Point (2013)	RPPr	29	29	1.00	24	0	0.413	0.417	0.048	This study
Parker Point (2014)	RPPr	31	31	1.00	24	0	0.465	0.446	−0.027	This study
Stark Bay (2013)	RSTr	27	27	1.00	27	0	0.545	0.498	−0.093	This study
Stark Bay (2014)	RSTr	31	31	1.00	30	0	0.510	0.519	0.042	This study
Thomson Bay (2013)	RTBr	34	34	1.00	34	1	0.454	0.444	−0.043	This study
Thomson Bay (2014)	RTBr	30	30	1.00	34	4	0.552	0.498	−0.101	This study

*Note*: Indices are based on the complete data set.

No recruits were located at Nancy Cove (RNC) 2013 and 2014.

^a^ All established meadows were in HWE when tests were performed using MLGs only.

* Significant deviation from HWE where *p* = .001.

### 3.5 Seed predation

**FIGURE 6:**
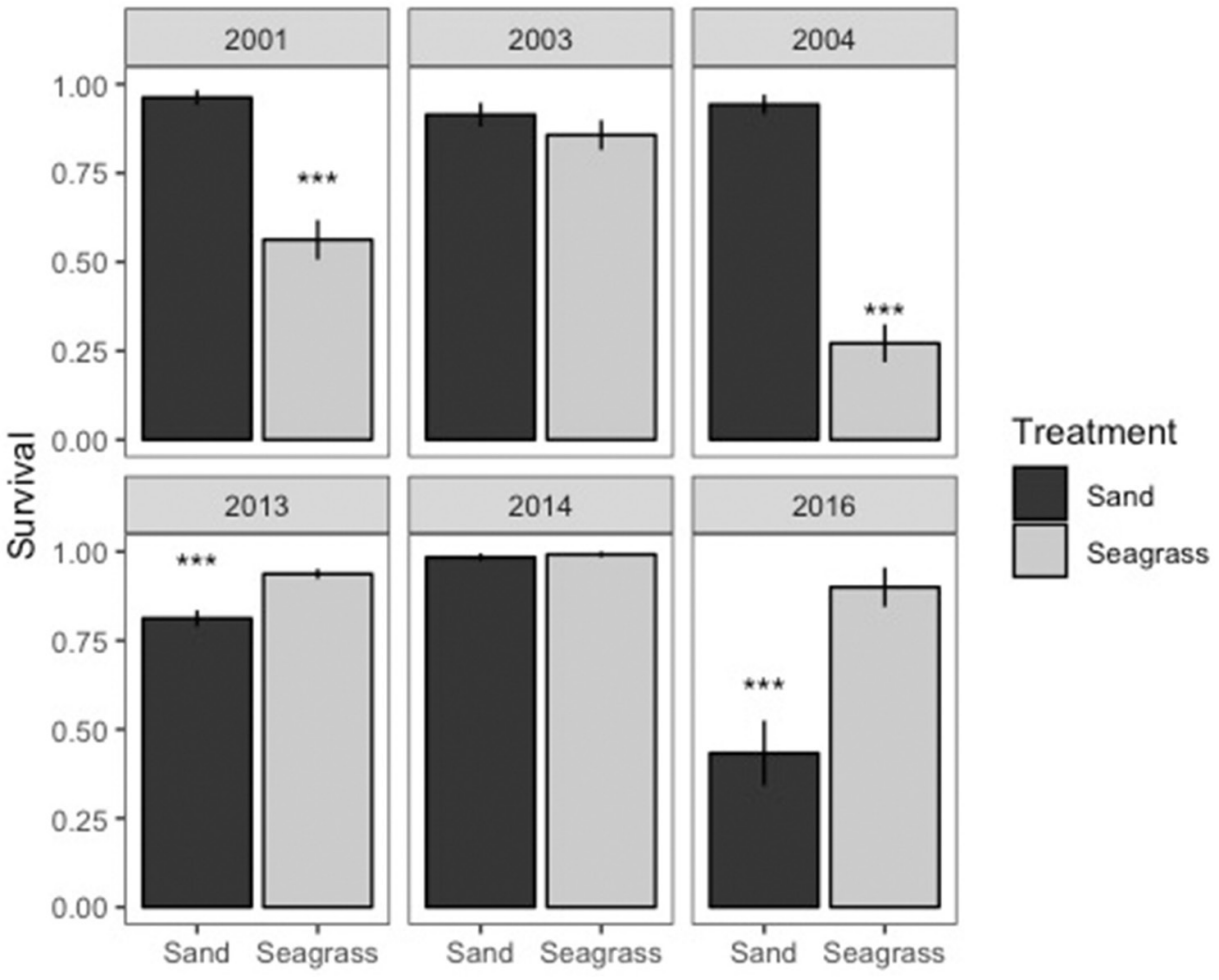
Proportional survival of tethered seeds between sand and seagrass habitats and among years over 15 years (2001, 2003, 2004, 2013, 2014, and 2016). Statistical significance using an analysis of deviance of a linear mixed model of presence or absence of grazing. ***p < .001.

Seed predation was dramatically different between the first 3 years (2001, 2003, 2004) and the latter 3 years (2013, 2014, and 2016) (Figure 6). Predation of seeds in 2001 and 2004 was significantly greater (*p* < .001) in seagrass compared with sand at Parker Point, except for 2003 where predation was not statistically different between seagrass and sand, although only seagrass habitats recorded seed loss. Predation patterns were reversed in 2013 and 2016, with higher rates in sand than seagrass habitats. Seed predation was low and not statistically different between seagrass and sand in 2014. Differences were significant based on analysis of deviance from a generalized linear mixed model. Distance from the meadow across all years was also generally significant (*p* < .001), so survival is expected to decrease with increasing distance (either away from or inside the meadow). We note that distance was only assessed for those years where predation over sand was greatest (2013, 2014, 2016). The interaction between seagrass or sand and distance from seagrass was not significant. Remote video camera observations of seed predation indicated a major shift in the presence of an omnivorous blue swimmer crab, *Portunus armatus*. We did not observe *P. armatus* at Parker Point between 2001 and 2004. However, between 2013 and 2016, we observed *P. armatus* consuming all seeds placed in sand. Another crab, *Nectocarcinus integrifrons*, was observed consuming seeds in seagrass between 2001 and 2004 but was not observed either consuming seeds or in sampling over seagrass between 2013 and 2016.

### 3.6 Recruitment

Seedlings survived and grew within all three surveyed locations from November 2014 to November 2018. A large number of seedlings were recruited at Parker Point in March 2015 (Figure S3), and almost half of the seedlings survived the first winter to November 2015. Similarly, older recruits recorded in November 2015 had grown and increased their number of shoots since counts in the previous March. Recruitment and recruit numbers increased every year at Parker Point until December 2018, when extensive boat mooring chain damage prompted a dramatic decline in older, larger recruits. We found fewer seedlings at Stark Bay (Figure S4) than Parker Point in 2014. However, their survival was high over the first year. Like Parker Point, large seedlings in Stark Bay declined in November 2018 after the realignment of the sandy patch from boat prop wash damage during summer and strong storm activity during winter. New seedling recruits appeared every year at Thomson Bay (Figure S5) and existing seedlings survived the duration of the trial. The age distribution was bimodal, with 3‐ or 4‐year‐old recruits (recruits with ~8 shoots) being fewer in number than 1–2‐year‐old recruits. Recruitment and growth of existing recruits continued, and this meadow has demonstrated significant recruitment from seeds over the 4 years.

Proportional survivorship was fitted to a power equation where survivors = number of initial recruits × age^mortality rate^ for ease of comparison among locations, although pseudo‐*R* ^2^ statistics indicated variable fit among locations with poorest fit from Stark Bay and significantly better fits from Thomson Bay and Parker Point (Figure S6, Table 3). The survivorship curves for seedling recruits in Stark Bay differed significantly in both initial recruit proportions and mortality rate from Parker Point and Thomson Bay (Figure S6, Table 3), with significantly lower proportion of 4 month recruits, and a lower rate of mortality among >1 year cohorts than the other two locations. Parker Point and Thomson Bay recruits were similar in both predicted initial recruit proportions, and in the slope in mortality, although there were slightly higher mortalities at Parker Point (Figure S6, Table 3).

**TABLE 3:** Nonlinear curve fitting statistics for survivorship curves fitted to a power curve (#survivors – *a* × age^b^) for recruiting populations at Parker Point, Thomson Bay, and Stark Bay between 2014 and 2018.

Location	Curve fit	*a*	*b*	*t*	*p*	Pseudo *R* ^2^	95% lower	95% upper
Parker Point	Power #survivors = *a* × age^b^	214.8 ± 28.1	−0.939 ± 0.072	*a* = 7.635 *b* = 0.072	*a* = <.001 *b* = <.001	.9029	*a* = 156.9 *b* = −1.087	*a* = 272.7 *b* = −0.791
Thomson Bay	Power #survivors = *a* × age^b^	197.9 ± 33.5	−0.712 ± 0.077	*a* = 5.898 *b* = −9.293	*a* = <.001 *b* = <.001	.7617	*a* = 129.5 *b* = −0.869	*a* = 266.3 *b* = −0.556
Stark Bay	Power #survivors = *a* × age^b^	72.4 ± 17.1	−0.486 ± 0.094	*a* = 17.124 *b* = 0.094	*a* = <.001 *b* = <.001	.4763	*a* = 37.5 *b* = −0.676	*a* = 107.3 *b* = −0.295

*Note*: Pseudo‐*R*‐squared statistic calculated using the Nagelkerke (Cragg and Uhler) method.

### 3.7 Origin of seedlings

All “seeds of known origin” from Parker Point and Stark Bay were correctly assigned to their local meadow of origin when assignment was performed by cohort (Table 4), providing confidence for the assignment for seedling cohorts. Seedling cohorts from Thomson Bay, Stark Bay and Parker Point assigned to their local meadow in 2013 and 2014 (Table 4). Individual assignment for “seeds of known origin” remained high at Stark Bay (91%) but was much lower at Parker Point (29%) with eight seeds assigning to nonlocal seedlings. Stark Bay had a consistently higher number of seedlings assigned with confidence to the local meadow (85 and 65%) than Thompson Bay (54 and 30%) and Parker Point (31 and 23%) over the 2 years. Overall, low numbers of individual recruits were assigned with confidence to a nonlocal meadow, with the highest number identified at Parker Point.

**TABLE 4:** Results of the population assignment test outcomes to “Self” or “Other” sampled *P. australis* meadows from one sampled meadow and the columns indicate the meadow to which samples (MLGs) were “assigned” (genotypes had the highest likelihood of occurring).

Sampling site	Site code	*N*	Group assignment	Individual assign: Self (local meadow)	Individual assign: Other (nonlocal meadow)
Seeds of known origin (mating system)					
Stark Bay	RST	110	RST (100%)	100 (91%)	0
Parker Point	RPP	139	RPP (100%)	40 (29%)	8 (RTB, RST, RNC, CI)
Seedling recruits: 2013				Apply threshold of −log(L) > 1	
Thomson Bay	RTB	35	RTB (100%)	19 (54%)	0
Stark Bay	RST	27	RST (100%)	23 (85%)	1 (RNC)
Parker Point	RPP	29	RPP (100%)	9 (31%)	7 (RTB, RST, CI)
Seedling recruits: 2014					
Thomson Bay	RTB	30	RTB (100%)	9 (30%)	3 (RST, RPP)
Stark Bay	RST	31	RST (100%)	20 (65%)	1 (RNC)
Parker Point	RPP	31	RPP (100%)	7 (23%)	8 (RTB, RST)

Abbreviations: CI, Carnac Island (32 km SE); RNC, Nancy Cove; RPP, Parker Point; RST, Stark Bay; RTB, Thomson Bay.

## 4 DISCUSSION

The significant spatial and temporal variation in flowering, seed production, and seedling recruitment observed in the seagrass *Posidonia australis* is not unusual in seagrasses (Kendrick et al., 2017) and variation in reproduction has previously been linked to environmental (climate) change (e.g. Diaz‐Almela et al., 2007) and population genetics (e.g. Jahnke et al., 2015; Sinclair et al., 2020). Here, we demonstrate that for *P. australis* at Rottnest Island, differences in flowering seed production and seedling recruitment were associated with differences in seagrass clonal size and genetic diversity interacting with seasonal and annual differences in the strength of surface currents (Alaee et al., 2007). Seed settlement and seedling recruitment in northern bays were more localized as dispersing fruits (seeds) were trapped in the island wake formed on the northern shore during summer (Alaee et al., 2007). In contrast, strong surface currents on the southern shores increased transport of the floating fruits from other locations, consistent with a higher number of nonlocal genotypes observed in recruits at Parker Point. When seeds settled and seedlings developed, grazing was intense and between 36 and 85% of all settled seeds were consumed daily (Orth et al., 2006; this study). Disturbance by bioturbating sea urchins and sand dollars was reported as another major cause of seed and seedling mortality in *P. australis* (Johnson et al., 2018). Similar seed predation (Fishman & Orth, 1996) and burial by bioturbators (Blackburn & Orth, 2013) have been described for *Zostera marina* in the northern hemisphere. Despite these major recruitment bottlenecks, continuous recruitment occurred resulting in dynamic, multi‐aged, and genetically diverse meadows.

This study adds to existing knowledge in fruit and seed production (Campey et al., 2002), seed characteristics (Kendrick, Pomeroy, et al., 2019), seed viability (Sinclair et al., 2016) and seed dispersal (Ruiz‐Montoya et al., 2015) in endemic Australian *Posidonia* species. Seed densities for *P. australis* from Rottnest Island were similar to previous records of annual seed production from seagrasses with non‐dormant recalcitrant seeds: for example, 260 seeds m^−2^ (*Thalassia hemprichii*: Rollon et al., 2001) and; 680 seeds m^−2^ (*P. australis*: Cambridge & Hocking, 1997). The abundant annual seed production in *P. australis* contrasts with its congener, *P. oceanica* in the northern part of its geographical range in the Mediterranean except in cases of extreme ocean warming events (Marín‐Guirao et al., 2019).

Biological factors can operate as an environmental “sieve” to seedling recruitment (Eriksson & Ehrlén, 1992; Orth et al., 2006; Statton et al., 2017). High levels of predation were observed by a range of predominantly crustacean consumers with shifts from higher level of consumption in seagrass meadows (2001–2004) by the isopod, *Cymodoce* sp. and portunid crab *Nectocarcinus integrifrons* (Orth et al., 2006) to the portunid crab, *Portunus armatus* feeding on seeds in open sandy areas and not seagrass meadows (2013–2016). We hypothesize that this change in predators may be a result of a significant marine heat wave during the 2010–2011 summer that altered the distribution and abundance patterns of numerous marine species in Western Australia (Wernberg et al., 2016). These observations demonstrate that the predation bottleneck for successful seed and seedling survivorship can shift between years and habitats (e.g. seagrass and bare sand) depending on changes in predator guilds and is a major secondary influence on recruitment success under future climate change. This shift in seed consumers and their activities between seagrass and sand habitats before and after an extreme marine heat wave is further evidence that climate warming impacts the species composition of marine consumers (Whalen et al., 2020) and the architecture of trophic food webs (Nagelkerken et al., 2020). The 2010–2011 marine heat wave resulted in collapse of the seagrass dominated shallow seagrass banks of Shark Bay, some 1000 km north of Rottnest Island, that impacted major consumers (crab, turtle, dugong) and their predators (Kendrick, Nowicki, et al., 2019) reducing the capacity of the seagrass dominated ecosystem to adapt to continuing change.

Successful seed recruitment is heavily influenced by localized physical disturbances, such as the seasonal occurrence of storms (Olesen et al., 2004), sediment burial events (Blackburn & Orth, 2013), and habitat fragmentation (Vermaat et al., 2004). At Rottnest Island, different natural and anthropogenic drivers of loss occur under winter and summer conditions. During most winters, four to eight major storms in the southwest of Australia generate waves sufficient to scour recruiting seedlings from sand sheets (Statton et al., 2017) and damage established seagrass meadows. During summer, significant boat disturbance by propellor wash, anchor, and mooring damage of recruiting seedlings was observed at Rottnest Island in all locations, especially in 2018. Disturbance of seagrass meadows caused by boating has been well documented previously at Rottnest Island (Hastings et al., 1995) and other seagrass meadows, globally (Glasby & West, 2018; Sagerman et al., 2020). Despite these disturbances, gaps are constantly being colonized, with recruitment occurring annually, and adult losses are balanced by recruitment from seeds, clonal growth, and meadow infilling.

This study demonstrates the important role that annual sexual reproduction and recruitment can play to maintain resilience in seagrass meadows (Kendrick et al., 2017). *Posidonia australis* was able to flower and persist through a life history strategy that maximizes both clonal growth and recruitment from seeds, resulting in risk spreading in disturbed seagrass landscapes. The additive effect of climate induced ocean warming on this species is yet to be understood but this study will form a detailed baseline to assess climate‐driven changes in flowering phenology, seed set and recruitment in the future. The present study included ecological, genetic and oceanographic data, and more directly demonstrates that reproductive effort and recruitment from seeds for *P. australis* is a contemporary process that has the potential to maintain genetic diversity within local meadows. This process imparts a level of resilience to climate change from both a demographic and evolutionary perspective.

## AUTHOR CONTRIBUTIONS

**Gary A. Kendrick:** Conceptualization (lead); data curation (lead); formal analysis (lead); funding acquisition (lead); investigation (equal); methodology (equal); project administration (lead); resources (lead); software (equal); supervision (lead); validation (equal); visualization (equal); writing – original draft (lead); writing – review and editing (lead). **Marion L. Cambridge:** Conceptualization (supporting); data curation (supporting); formal analysis (supporting); funding acquisition (supporting); investigation (supporting); project administration (supporting); resources (supporting); writing – original draft (supporting); writing – review and editing (supporting). **Robert J. Orth:** Conceptualization (supporting); data curation (equal); formal analysis (equal); investigation (equal); methodology (equal); resources (equal); validation (equal); visualization (equal); writing – original draft (supporting); writing – review and editing (supporting). **Matthew W. Fraser:** Formal analysis (equal); investigation (supporting); methodology (supporting); validation (equal); visualization (equal); writing – review and editing (supporting). **Renae K. Hovey:** Formal analysis (supporting); investigation (supporting); software (supporting); visualization (equal); writing – review and editing (supporting). **John Statton:** Investigation (supporting); methodology (supporting); resources (supporting); writing – original draft (equal); writing – review and editing (supporting). **Charitha B. Pattiaratchi:** Formal analysis (supporting); investigation (supporting); methodology (supporting); visualization (supporting); writing – review and editing (supporting). **Elizabeth A. Sinclair:** Conceptualization (equal); data curation (equal); formal analysis (equal); funding acquisition (supporting); investigation (equal); methodology (equal); validation (equal); visualization (equal); writing – original draft (supporting); writing – review and editing (equal).

## Supporting information


Appendix S1.

## Data Availability

Data are available from Dryad for Flowering, seed production, predation, and recruitment (DOI: 10.5061/dryad.vhhmgqnwz) and Microsatellite genotypes from adult and seedlings of the temperate seagrass (ribbon weed) *Posidonia australis*, from four meadows at Rottnest Island, Western Australia (DOI: 10.5061/dryad.dfn2z3577).
