# Supplementary material for: The cycle of seagrass life: From flowers to new meadows
Source: Ecol Evol. 2023 Aug 31;13(9):e10456. doi: 10.1002/ece3.10456 (PMC10469021; doi:10.1002/ece3.10456)

**SUPPORTING INFORMATION**

**TABLE S1** Estimates, Standard Error, Z values and probabilities for the fixed effects location, year and interaction location x year for the response variables shoot density, inflorescence density, flower density and seed density (individuals m^-2^). Comparisons to Parker Point.

| Shoot Density: Poisson (log) |  |  |  |  |  |
| --- | --- | --- | --- | --- | --- |
|  | Estimate | Std. Error | z value | Pr(>\|z\|) |  |
| (Intercept) | 6.27792 | 0.06785 | 92.521 | < 2e-16 | *** |
| locationNancy Cove | -0.64384 | 0.09773 | -6.588 | 4.45E-11 | *** |
| locationStark Bay | 0.3585 | 0.09537 | 3.759 | 0.000171 | *** |
| locationCrayfish Rock | 0.21704 | 0.09563 | 2.27 | 0.023232 | * |
| locationCatherine Pt | -0.20733 | 0.09644 | -2.15 | 0.031575 | * |
| locationThomson Bay | -0.06365 | 0.09612 | -0.662 | 0.507823 |  |
| year2014 | -0.36506 | 0.09683 | -3.77 | 0.000163 | *** |
| year2015 | -0.29458 | 0.09662 | -3.049 | 0.002298 | ** |
| year2016 | -0.2851 | 0.09662 | -2.951 | 0.003169 | ** |
| year2017 | -0.04179 | 0.09605 | -0.435 | 0.663509 |  |
| year2018 | -0.08622 | 0.09613 | -0.897 | 0.369788 |  |
| locationNancy Cove:year2014 | 0.59716 | 0.13827 | 4.319 | 1.57E-05 | *** |
| locationStark Bay:year2014 | 0.15518 | 0.13572 | 1.143 | 0.252868 |  |
| locationCrayfish Rock:year2014 | 0.50115 | 0.13568 | 3.694 | 0.000221 | *** |
| locationCatherine Pt:year2014 | 0.56237 | 0.13669 | 4.114 | 3.88E-05 | *** |
| locationThomson Bay:year2014 | 0.36896 | 0.13653 | 2.702 | 0.006882 | ** |
| locationNancy Cove:year2015 | 0.56209 | 0.13805 | 4.072 | 4.67E-05 | *** |
| locationStark Bay:year2015 | 0.1038 | 0.13555 | 0.766 | 0.443827 |  |
| locationCrayfish Rock:year2015 | 0.53655 | 0.13544 | 3.961 | 7.45E-05 | *** |
| locationCatherine Pt:year2015 | 0.58616 | 0.13643 | 4.296 | 1.74E-05 | *** |
| locationThomson Bay:year2015 | 0.31011 | 0.13636 | 2.274 | 0.022959 | * |
| locationNancy Cove:year2016 | 0.42107 | 0.13832 | 3.044 | 0.002333 | ** |
| locationStark Bay:year2016 | -0.03461 | 0.13572 | -0.255 | 0.798696 |  |
| locationCrayfish Rock:year2016 | 0.37952 | 0.13557 | 2.799 | 0.005119 | ** |
| locationCatherine Pt:year2016 | 0.69339 | 0.13627 | 5.088 | 3.61E-07 | *** |
| locationThomson Bay:year2016 | 0.05881 | 0.13676 | 0.43 | 0.667201 |  |
| locationNancy Cove:year2017 | 0.41624 | 0.13745 | 3.028 | 0.002458 | ** |
| locationStark Bay:year2017 | 0.01167 | 0.13497 | 0.086 | 0.931101 |  |
| locationCrayfish Rock:year2017 | 0.60841 | 0.13479 | 4.514 | 6.36E-06 | *** |
| locationCatherine Pt:year2017 | 0.43446 | 0.1359 | 3.197 | 0.001389 | ** |
| locationThomson Bay:year2017 | 0.30098 | 0.13565 | 2.219 | 0.026501 | * |
| locationNancy Cove:year2018 | 0.54916 | 0.13735 | 3.998 | 6.38E-05 | *** |
| locationStark Bay:year2018 | -0.13682 | 0.13525 | -1.012 | 0.311748 |  |
| locationCrayfish Rock:year2018 | 0.32361 | 0.13509 | 2.396 | 0.016593 | * |
| locationCatherine Pt:year2018 | 0.63422 | 0.13577 | 4.671 | 2.99E-06 | *** |
| locationThomson Bay:year2018 | 0.35717 | 0.13568 | 2.632 | 0.008478 | ** |
|  |  |  |  |  |  |
| Inflorescence density: Poisson (log) |  |  |  |  |  |
|  | Estimate | Std. Error | z value | Pr(>\|z\|) |  |
| (Intercept) | 3.5081 | 0.2375 | 14.772 | < 2e-16 | *** |
| locationNancy Cove | -0.4485 | 0.3421 | -1.311 | 0.189853 |  |
| locationStark Bay | 2.3161 | 0.3262 | 7.1 | 1.24E-12 | *** |
| locationCrayfish Rock | 2.534 | 0.3261 | 7.77 | 7.86E-15 | *** |
| locationCatherine Pt | 1.6756 | 0.3272 | 5.121 | 3.04E-07 | *** |
| locationThomson Bay | 1.5246 | 0.3274 | 4.657 | 3.21E-06 | *** |
| year2014 | -1.2173 | 0.3601 | -3.38 | 0.000724 | *** |
| year2015 | -0.5229 | 0.3405 | -1.536 | 0.124645 |  |
| year2016 | -0.1471 | 0.3366 | -0.437 | 0.662054 |  |
| year2017 | 0.9294 | 0.329 | 2.825 | 0.004726 | ** |
| year2018 | 0.1871 | 0.3328 | 0.562 | 0.573958 |  |
| locationNancy Cove:year2014 | 0.3916 | 0.5112 | 0.766 | 0.443551 |  |
| locationStark Bay:year2014 | 0.2139 | 0.4804 | 0.445 | 0.656095 |  |
| locationCrayfish Rock:year2014 | 0.1391 | 0.4804 | 0.29 | 0.77217 |  |
| locationCatherine Pt:year2014 | 0.1785 | 0.4838 | 0.369 | 0.712149 |  |
| locationThomson Bay:year2014 | 1.1071 | 0.4811 | 2.301 | 0.021378 | * |
| locationNancy Cove:year2015 | 0.4488 | 0.486 | 0.923 | 0.355803 |  |
| locationStark Bay:year2015 | 1.1819 | 0.4644 | 2.545 | 0.010926 | * |
| locationCrayfish Rock:year2015 | 1.4575 | 0.4642 | 3.14 | 0.001692 | ** |
| locationCatherine Pt:year2015 | 1.6233 | 0.4652 | 3.489 | 0.000484 | *** |
| locationThomson Bay:year2015 | 1.689 | 0.4654 | 3.63 | 0.000284 | *** |
| locationNancy Cove:year2016 | 0.8449 | 0.4778 | 1.768 | 0.076989 | . |
| locationStark Bay:year2016 | 0.3841 | 0.4618 | 0.832 | 0.405562 |  |
| locationCrayfish Rock:year2016 | 0.1724 | 0.4617 | 0.373 | 0.708905 |  |
| locationCatherine Pt:year2016 | 1.0611 | 0.4625 | 2.294 | 0.021782 | * |
| locationThomson Bay:year2016 | 0.7784 | 0.4628 | 1.682 | 0.0926 | . |
| locationNancy Cove:year2017 | -1.4419 | 0.4843 | -2.977 | 0.00291 | ** |
| locationStark Bay:year2017 | -0.5648 | 0.4561 | -1.238 | 0.215572 |  |
| locationCrayfish Rock:year2017 | -0.838 | 0.4561 | -1.837 | 0.066153 | . |
| locationCatherine Pt:year2017 | -2.0002 | 0.4603 | -4.345 | 1.39E-05 | *** |
| locationThomson Bay:year2017 | -0.4723 | 0.4576 | -1.032 | 0.301955 |  |
| locationNancy Cove:year2018 | 0.2522 | 0.4767 | 0.529 | 0.596692 |  |
| locationStark Bay:year2018 | -0.4466 | 0.4593 | -0.972 | 0.330954 |  |
| locationCrayfish Rock:year2018 | -0.7621 | 0.4596 | -1.658 | 0.097299 | . |
| locationCatherine Pt:year2018 | -0.4734 | 0.4609 | -1.027 | 0.304352 |  |
| locationThomson Bay:year2018 | 0.6924 | 0.4599 | 1.506 | 0.132181 |  |
|  |  |  |  |  |  |

Flower density:Negative Binomial

|  | Estimate | Std.Error | z-value | Pr(>\|z\|) |  |
| --- | --- | --- | --- | --- | --- |
| (Intercept) | 4.406739 | 0.091205 | 48.317 | < 2e-16 | *** |
| locationNancyCove | -0.027693 | 0.128770 | -0.215 | 0.829723 |  |
| locationStarkBay | 2.687772 | 0.125253 | 21.459 | < 2e-16 | *** |
| locationCrayfishRock | 2.634380 | 0.125269 | 21.030 | < 2e-16 | *** |
| locationCatherinePt | 1.933178 | 0.125576 | 15.394 | < 2e-16 | *** |
| locationThomsonBay | 1.773107 | 0.125666 | 14.110 | < 2e-16 | *** |
| year2014 | -0.900180 | 0.134611 | -6.687 | 2.27e-11 | *** |
| year2015 | -0.715773 | 0.133035 | -5.380 | 7.44e-08 | *** |
| year2016 | -0.323569 | 0.130483 | -2.480 | 0.013146 | * |
| year2017 | 0.105036 | 0.128595 | 0.817 | 0.414046 |  |
| year2018 | 0.040800 | 0.128818 | 0.317 | 0.751449 |  |
| locationNancyCove:year2014 | -0.491505 | 0.194642 | -2.525 | 0.011564 | * |
| locationStarkBay:year2014 | 0.034833 | 0.181536 | 0.192 | 0.847835 |  |
| locationCrayfishRock:year2014 | 0.073469 | 0.185620 | 0.396 | 0.692250 |  |
| locationCatherinePt:year2014 | 0.210993 | 0.182118 | 1.159 | 0.246638 |  |
| locationThomsonBay:year2014 | 0.863234 | 0.185932 | 4.643 | 3.44e-06 | *** |
| locationNancyCove:year2015 | 0.080330 | 0.187723 | 0.428 | 0.668712 |  |
| locationStarkBay:year2015 | 0.673838 | 0.180112 | 3.741 | 0.000183 | *** |
| locationCrayfishRock:year2015 | 0.891302 | 0.180096 | 4.949 | 7.46e-07 | *** |
| locationCatherinePt:year2015 | 1.395078 | 0.180350 | 7.735 | 1.03e-14 | *** |
| locationThomsonBay:year2015 | 1.399816 | 0.180446 | 7.758 | 8.66e-15 | *** |
| locationNancyCove:year2016 | 0.608749 | 0.182689 | 3.332 | 0.000862 | *** |
| locationStarkBay:year2016 | 0.232125 | 0.178252 | 1.302 | 0.192840 |  |
| locationCrayfishRock:year2016 | 0.302413 | 0.178257 | 1.697 | 0.089791 | . |
| locationCatherinePt:year2016 | 0.741650 | 0.178539 | 4.154 | 3.27e-05 | *** |
| locationThomsonBay:year2016 | 0.468976 | 0.178746 | 2.624 | 0.008698 | ** |
| locationNancyCove:year2017 | -1.042063 | 0.186452 | -5.589 | 2.29e-08 | *** |
| locationStarkBay:year2017 | 0.002122 | 0.176837 | 0.012 | 0.990424 |  |
| locationCrayfishRock:year2017 | 0.038349 | 0.176851 | 0.217 | 0.828328 |  |
| locationCatherinePt:year2017 | -1.350689 | 0.178331 | -7.574 | 3.62e-14 | *** |
| locationThomsonBay:year2017 | 0.214779 | 0.177307 | 1.211 | 0.225765 |  |
| locationStarkBay:year2018 | -1.062074 | 0.177360 | -5.988 | 2.12e-09 | *** |
| locationCrayfishRock:year2018 | -0.269943 | 0.177090 | -1.524 | 0.127428 |  |
| locationCatherinePt:year2018 | -0.740583 | 0.177884 | -4.163 | 3.14e-05 | *** |
| locationThomsonBay:year2018 | 0.487029 | 0.177399 | 2.745 | 0.006044 | ** |

Seed Density: Negative Binomial

|  | Estimate | Std.Error | z-value | Pr(>\|z\|) |  |
| --- | --- | --- | --- | --- | --- |
| (Intercept) | 4.406739 | 0.091205 | 48.317 | < 2e-16 | *** |
| locationNancyCove | -0.027693 | 0.128770 | -0.215 | 0.829723 |  |
| locationStarkBay | 2.687772 | 0.125253 | 21.459 | < 2e-16 | *** |
| locationCrayfishRock | 2.634380 | 0.125269 | 21.030 | < 2e-16 | *** |
| locationCatherinePt | 1.933178 | 0.125576 | 15.394 | < 2e-16 | *** |
| locationThomsonBay | 1.773107 | 0.125666 | 14.110 | < 2e-16 | *** |
| year2014 | -0.900180 | 0.134611 | -6.687 | 2.27e-11 | *** |
| year2015 | -0.715773 | 0.133035 | -5.380 | 7.44e-08 | *** |
| year2016 | -0.323569 | 0.130483 | -2.480 | 0.013146 | * |
| year2017 | 0.105036 | 0.128595 | 0.817 | 0.414046 |  |
| year2018 | 0.040800 | 0.128818 | 0.317 | 0.751449 |  |
| locationNancyCove:year2014 | -0.491505 | 0.194642 | -2.525 | 0.011564 | * |
| locationStarkBay:year2014 | 0.034833 | 0.181536 | 0.192 | 0.847835 |  |
| locationCrayfishRock:year2014 | 0.073469 | 0.185620 | 0.396 | 0.692250 |  |
| locationCatherinePt:year2014 | 0.210993 | 0.182118 | 1.159 | 0.246638 |  |
| locationThomsonBay:year2014 | 0.863234 | 0.185932 | 4.643 | 3.44e-06 | *** |
| locationNancyCove:year2015 | 0.080330 | 0.187723 | 0.428 | 0.668712 |  |
| locationStarkBay:year2015 | 0.673838 | 0.180112 | 3.741 | 0.000183 | *** |
| locationCrayfishRock:year2015 | 0.891302 | 0.180096 | 4.949 | 7.46e-07 | *** |
| locationCatherinePt:year2015 | 1.395078 | 0.180350 | 7.735 | 1.03e-14 | *** |
| locationThomsonBay:year2015 | 1.399816 | 0.180446 | 7.758 | 8.66e-15 | *** |
| locationNancyCove:year2016 | 0.608749 | 0.182689 | 3.332 | 0.000862 | *** |
| locationStarkBay:year2016 | 0.232125 | 0.178252 | 1.302 | 0.192840 |  |
| locationCrayfishRock:year2016 | 0.302413 | 0.178257 | 1.697 | 0.089791 | . |
| locationCatherinePt:year2016 | 0.741650 | 0.178539 | 4.154 | 3.27e-05 | *** |
| locationThomsonBay:year2016 | 0.468976 | 0.178746 | 2.624 | 0.008698 | ** |
| locationNancyCove:year2017 | -1.042063 | 0.186452 | -5.589 | 2.29e-08 | *** |
| locationStarkBay:year2017 | 0.002122 | 0.176837 | 0.012 | 0.990424 |  |
| locationCrayfishRock:year2017 | 0.038349 | 0.176851 | 0.217 | 0.828328 |  |
| locationCatherinePt:year2017 | -1.350689 | 0.178331 | -7.574 | 3.62e-14 | *** |
| locationThomsonBay:year2017 | 0.214779 | 0.177307 | 1.211 | 0.225765 |  |
| locationStarkBay:year2018 | -1.062074 | 0.177360 | -5.988 | 2.12e-09 | *** |
| locationCrayfishRock:year2018 | -0.269943 | 0.177090 | -1.524 | 0.127428 |  |
| locationCatherinePt:year2018 | -0.740583 | 0.177884 | -4.163 | 3.14e-05 | *** |
| locationThomsonBay:year2018 | 0.487029 | 0.177399 | 2.745 | 0.006044 | ** |

**TABLE S2** Location, dates, duration of experiments and methodology used to assess seed predation on Rottnest Island, Western Australia.

| Year | Location | Date | Days | Methodology | |
| --- | --- | --- | --- | --- | --- |
| 2001 | Parker Point | Nov. 21-28 | 7 | Tethers | |
|  | Stark Bay | Nov. 25-29 | 4 | Tethers | |
| 2003 | Parker Point | Nov. 21-28 | 7 | Tethers | |
|  | Nancy Cove | Nov. 25-28 | 3 | Tethers | |
| 2004 | Parker Point | Nov. 21-28 | 7 | Tethers | |
|  | Nancy Cove | Nov. 21-28 | 7 | Tethers | |
|  | Stark Bay | Nov. 21-28 | 7 | Tethers | |
|  | Catherine Bay | Nov. 21-28 | 7 | Tethers | |
|  | Parakeet Bay | Nov. 21-28 | 7 | Tethers | |
| 2013 | Parker Point | Nov. 20-28 | 8 | Seed boards | |
|  | Stark Bay | Nov. 25-27 | 2 | Seed boards | |
| 2014 | Parker Point | Nov. 20-27 | 7 | Seed boards |  |
|  | Nancy Cove | Nov. 20-27 | 7 | Tethers | |
|  | Stark Bay | Nov. 22-28 | 6 | Tethers and seed boards | |
|  | Catherine Bay | Nov. 22-28 | 6 | Tethers | |
| 2016 | Parker Point | Nov. 22-24 | 3 | Tethers | |

**SUPPLEMENTARY FIGURES**


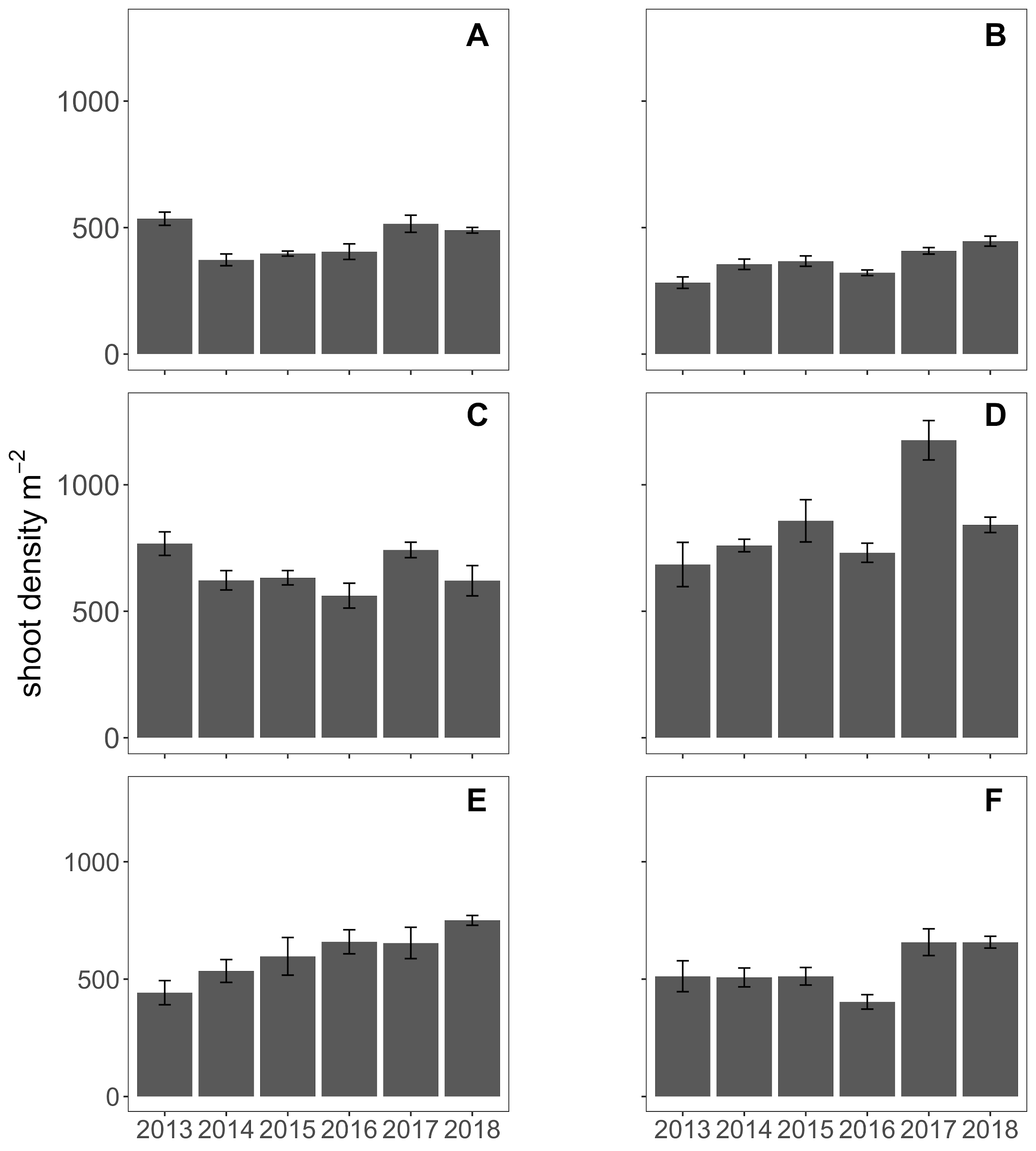


**FIGURE S1** Vegetative shoot densities (m^2^ ± SE, n = 10) measured annually in November from 2013 to 2018 for the six locations: A) Parker Point, B) Nancy Cove, C) Stark Bay, D) Crayfish Rock, E) Catherine Point and F) Thomson Bay at Rottnest Island.


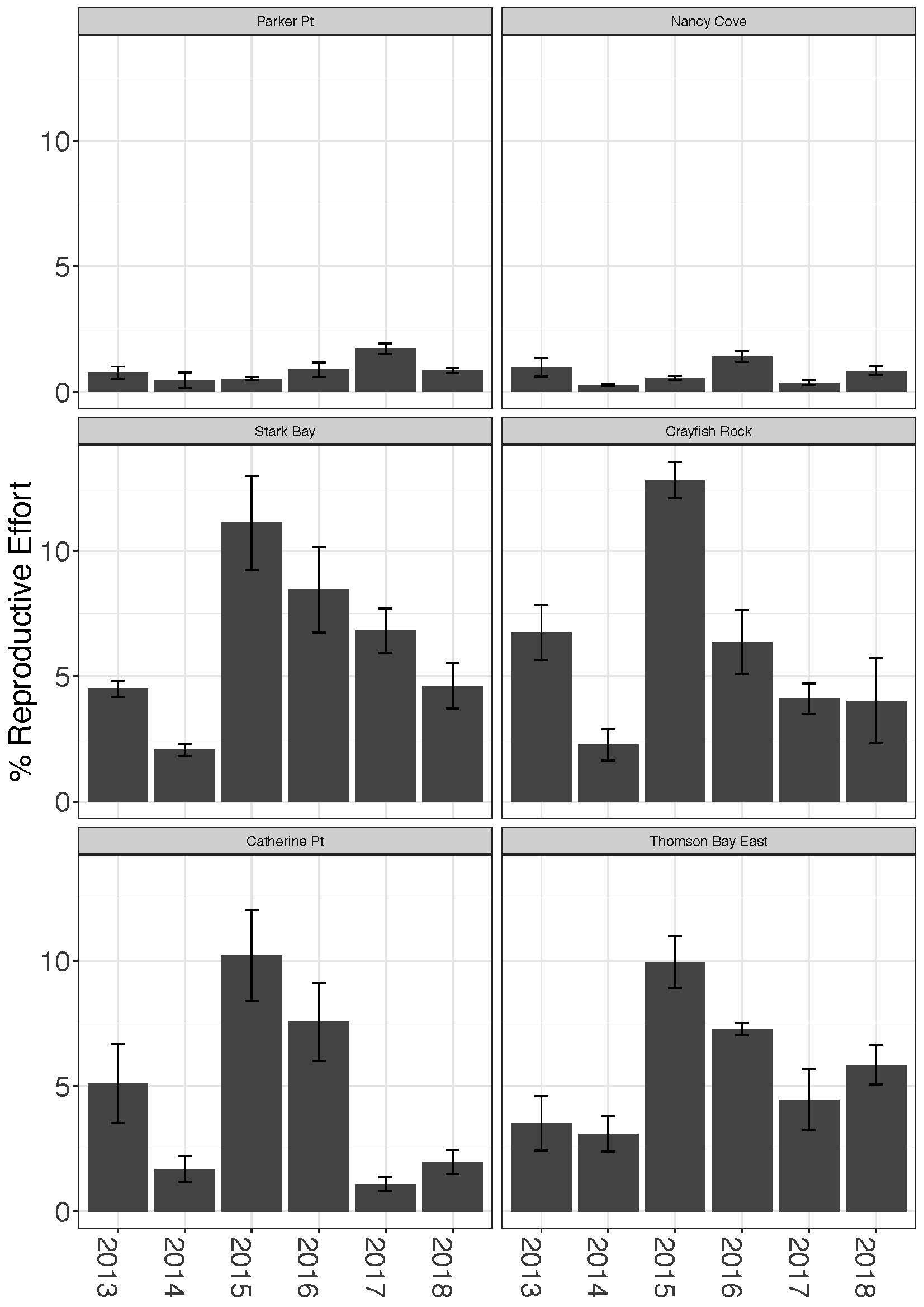


**FIGURE S2** Reproductive Effort (inflorescence density/shoot density, mean ± SE, n = 10) measured annually in November from 2013 to 2018 for the six locations: A) Parker Point, B) Nancy Cove, C) Stark Bay, D) Crayfish Rock, E) Catherine Point and F) Thomson Bay at Rottnest Island.


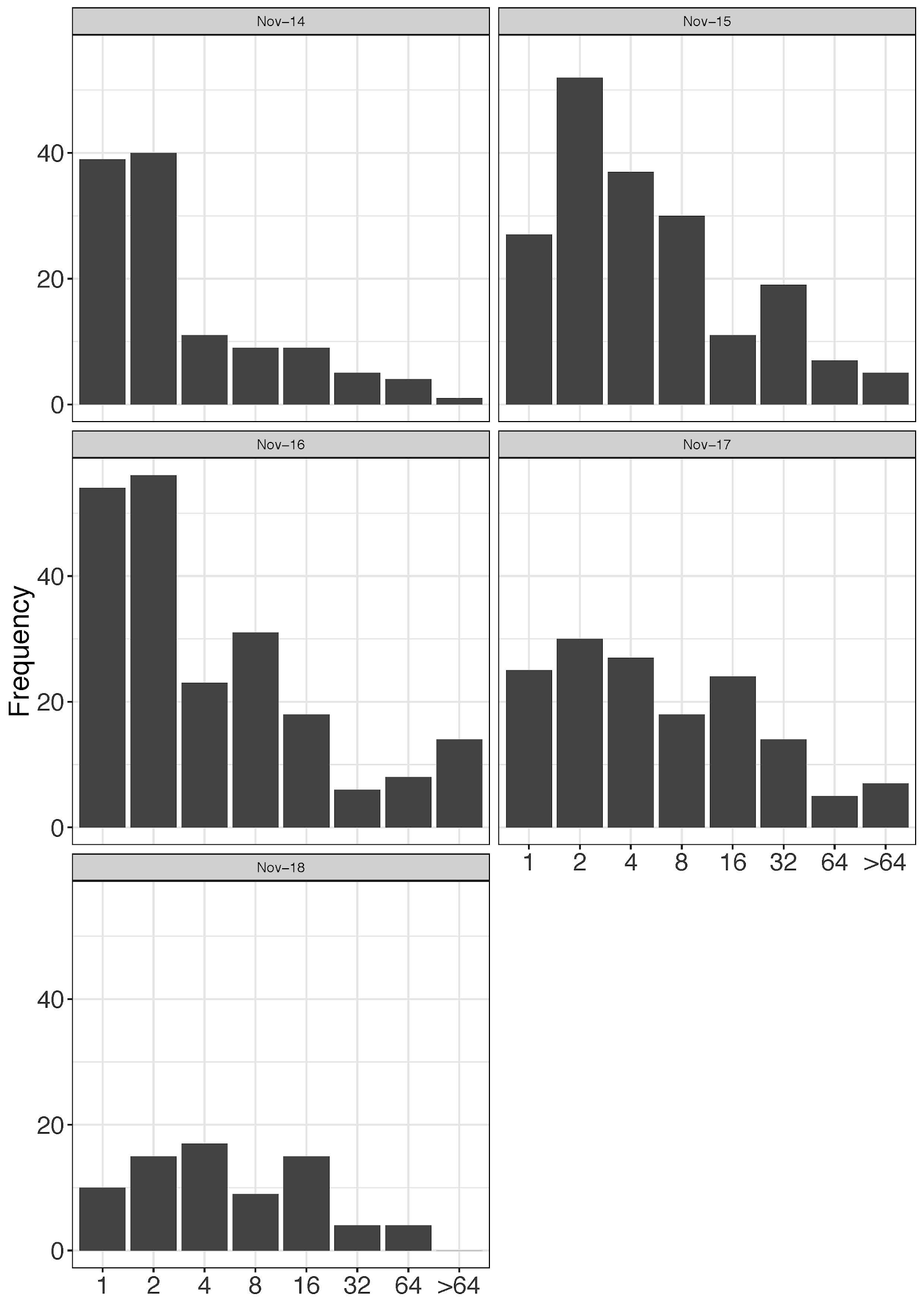


**FIGURE S3** Frequency histograms of the number of recruits, binned by 1, 2, 4, 8, 16, 32, 64, >64 shoots, found in a single 300 m area at Parker Point from November 2014 to 2018.


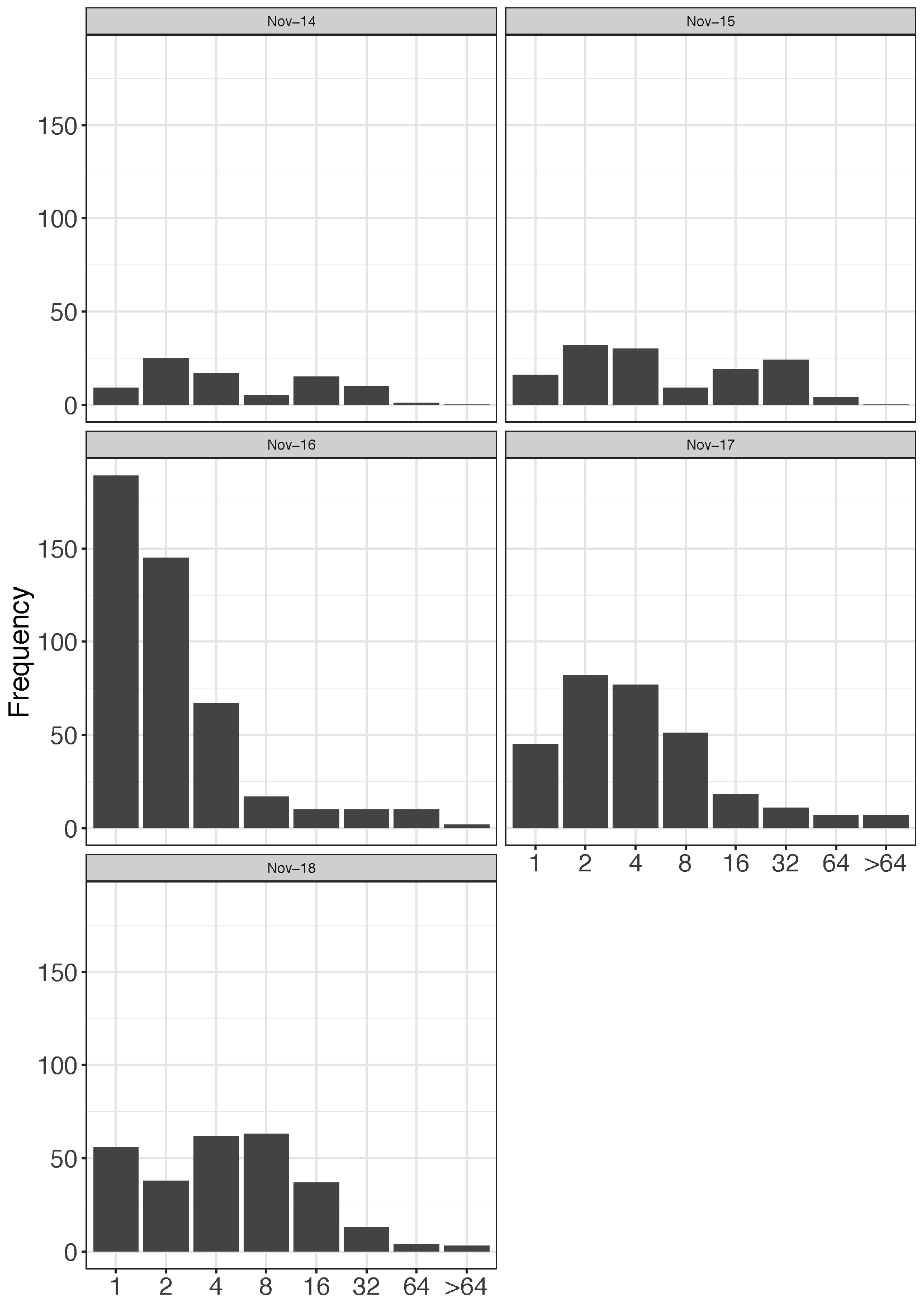


**FIGURE S4** Frequency histograms of the number of recruits, binned by 1, 2, 4, 8, 16, 32, 64, >64 shoots, found in a single 300 m area at Thomson Bay from November 2014 to 2018.

**
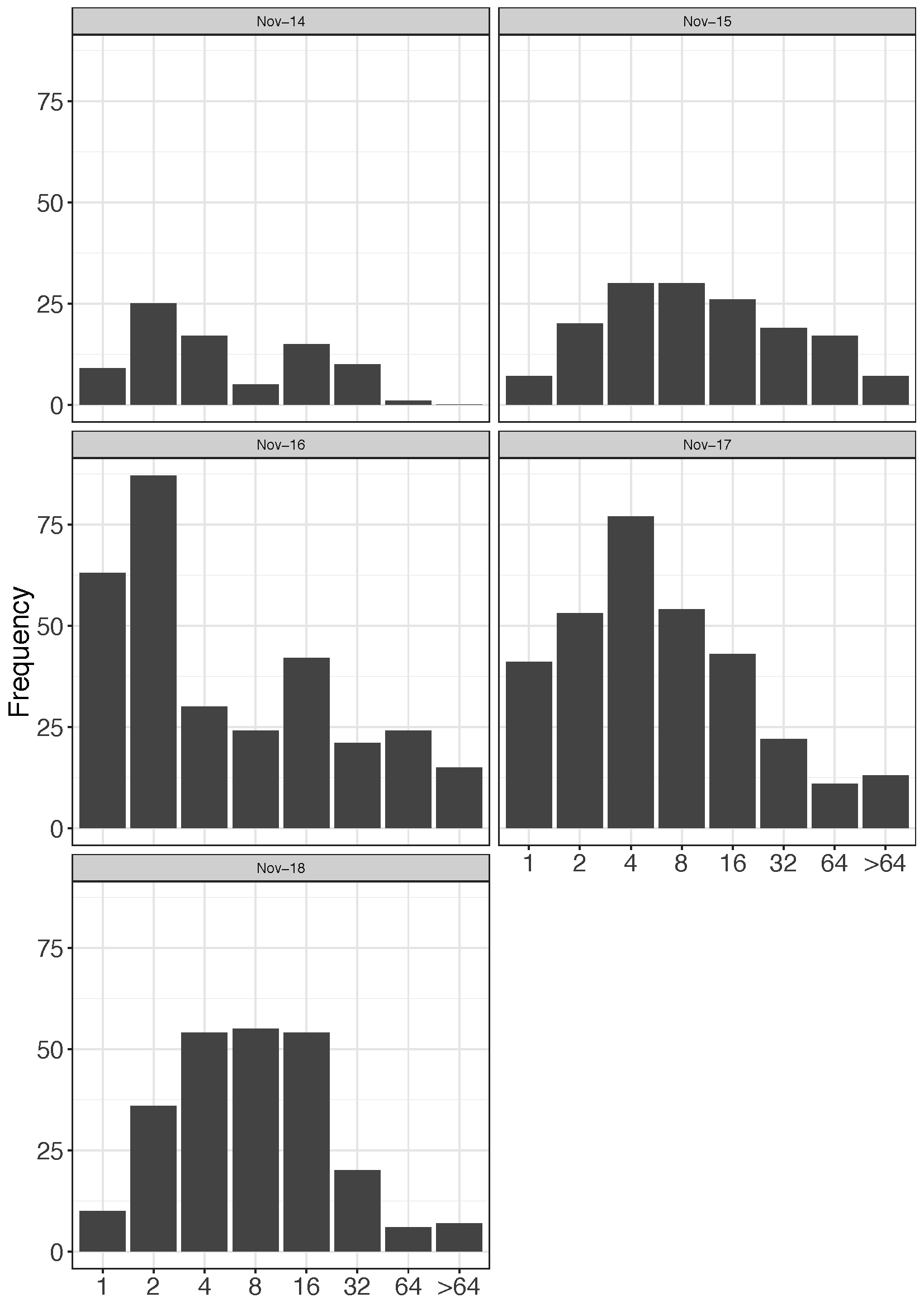
**

**FIGURE S5** Frequency histograms of the number of recruits, binned by 1, 2, 4, 8, 16, 32, 64, >64 shoots, found in a single 300 m area at Stark Bay from November 2014 to 2018.

**FIGURE S6** Survivorship curves for A) Thomson Bay, B) Parker Point Anchorage and C) Stark Bay determined from the proportion of each age class (months).
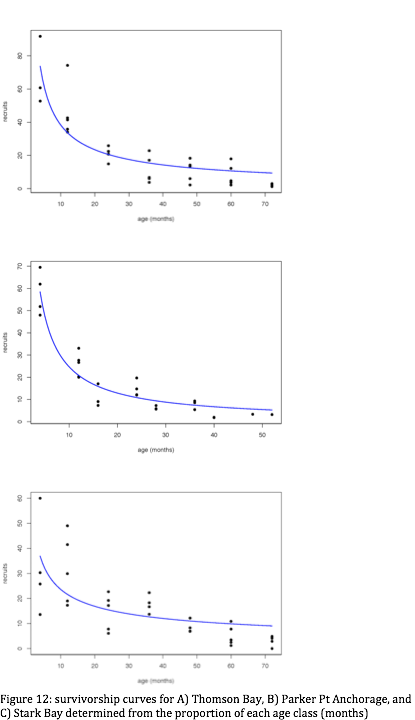

Supplement: Supplementary file 1 — Appendix S1. [file ECE3-13-e10456-s001.docx]
